# An Advanced Sensing Approach to Biological Toxins with Localized Surface Plasmon Resonance Spectroscopy Based on Their Unique Protein Quaternary Structures

**DOI:** 10.3390/ijms252413352

**Published:** 2024-12-12

**Authors:** Hirotaka Uzawa, Satoshi Kondo, Takehiro Nagatsuka, Yasuo Seto, Yoshihiro Nishida

**Affiliations:** 1 Nanomaterials Research Institute, National Institute of Advanced Industrial Science and Technology (AIST), 1-1-1 Higashi, Tsukuba 305-8565, Ibaraki, Japan; 2 Forensic Science Group, RIKEN SPring-8 Center, Sayo-gun 679-5148, Hyogo, Japan; seto.y@spring8.or.jp; 3 Division of Applied Biological Chemistry, Graduate School of Environmental Horticulture, Chiba University, Matsudo 271-8510, Chiba, Japan; ynishida775@gmail.com

**Keywords:** localized surface plasmon resonance (LSPR) biosensor, glycolipid chips, multivalent antibody-Au nanoconjugates, AB toxins, botulinum neurotoxin, ricin

## Abstract

Botulinum neurotoxins (BoNTs), ricin, and many other biological toxins are called AB toxins possessing heterogeneous A and B subunits. We propose herein a quick and safe sensing approach to AB toxins based on their unique quaternary structures. The proposed approach utilizes IgG antibodies against their A-subunits in combination with those human cell-membrane glycolipids that act as the natural ligands of B-subunits. In practice, an IgG antibody against the A-subunit of a target toxin is selected from commercially available sources and immobilized on the surface of Au nanoparticles to constitute a multivalent IgG/Au nanoconjugate. The derived IgG/Au conjugate is used in the pretreatment process of test samples for deactivating biological toxins in the form of a ternary toxin/antibody/Au complex. This process is implemented in advance to reduce the risk of handling biological toxins in laboratory work. On the other hand, the human glycolipid is immobilized on a tiny glass plate and used as a biosensor chip. The biosensor chip is set in the chamber of a flow sensing system using localized surface plasmon resonance (LSPR) spectrometry available in portable size at relatively low cost. In principle, the LSPR sensing system enables us to perform a rapid and selective detection for different kinds of biological toxins if the human glycolipid is correctly selected and installed in the sensing system. In the present LSPR sensing approach, a target AB toxin may have been deactivated during the pretreatment process. The test sample containing the deactivated AB toxin becomes a real target to be analyzed by the sensing system. In the present, we describe the concept of employing the commercially available IgG antibody in the pretreatment process followed by a typical procedure for converting it into the multivalent antibody/Au nanoconjugate and its preliminary applications in the LSPR detection of a ricin homologue (RCA_120_) and BoNTs in different serotypes. The tested LSPR sensing approach has worked very well for the ricin homologue and certain serotypes of botulinum neurotoxins like BoNT/A, indicating that the prior deactivation process at their A-domains causes no significant damage to the function of their B-domains with respect to determining the host cell-membrane glycolipid. The experimental results also indicated that LSPR responses from these pretreated AB toxins are significantly amplified. That is obviously thanks to the presence of Au nanoparticles in the multivalent IgG/Au nanoconjugate. We suggest in conclusion that the proposed LSPR sensing approach will provide us with a safe and useful tool for the study of biological AB toxins based on their unique quaternary protein structures.

## 1. Introduction

**Figure 1:**
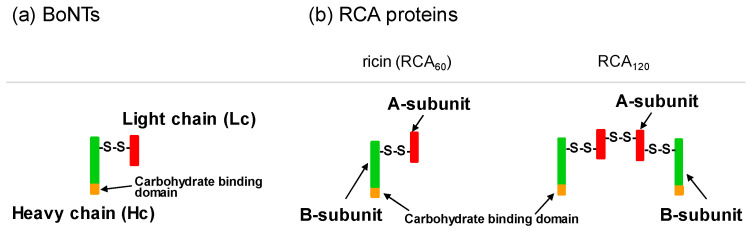
A representation of biological AB toxins: (**a**) BoNTs and (**b**) ricin (RCA_60_) and RCA_120_, in which molecular shapes and sizes are roughly shown.

Botulinum neurotoxins (BoNTs) from *Clostridium botulinum* strains constitute seven different serotypes (type A to G) and more than 40 subtypes [1,2]. They are thought to be among the most potent toxins on earth. An estimated human lethal dose (LD_50_) of BoNT/A is reported to be 1.3~2.1 ng/kg on intramuscular injection, 10~13 ng/kg on inhalation, and 1 μg/kg on oral administration [3,4]. BoNTs are listed in category A pathogens by Centers for Disease Control and Prevention (CDC) [5]. Their strong neurotoxicity originates in a simple and single peptide chain (~150 kDa), which is proteolyzed into light (Lc; 50 kDa) and heavy (Hc; 100 kDa) chains (Figure 1a) [4]. The Lc chain corresponds to the enzymatic A-domain of the AB type of biological toxin: this domain enables BoNTs to cleave to soluble *N*-ethylmaleimide-sensitive factor attachment protein receptor (SNARE) proteins and block the release of the neurotransmitter acetylcholine, causing neural disorders. The heavy chain (Hc) corresponds to the B domain of AB toxins and possesses a pair of receptor binding sites: one is for binding with sialyl oligosaccharides (gangliosides like GT1b and GD1b) expressed on neurocyte membranes, and the other for a receptor component (synaptic vesicle protein 2) SV2 [6,7]. The heavy chains assist BoNTs in causing endocytosis and translocation by maintaining close contact with neurocyte membranes. Native BoNTs have variations in molecular weights (150~900 kDa) by complexation with other proteins such as hemagglutinins (HAs) and non-toxic non-hemagglutinin (NTNHA) at the Lc chain [4].

An analogous AB structure can be seen in ricin (RCA_60_, Figure 1b), which is a botanical toxin occurring in seeds of *Ricinus communis*. Its A-domain has an RNA ribonuclease activity, while a B-domain has a pair of receptor binding sites. Different from BoNTs, the receptor binding sites recognize *exo* terminal β-Gal and β-GalNAc residues in glycoproteins or glycolipids with affinities different from one another [8,9,10]. *R. communis* also produces an agglutinin (RCA_120_), which is a dimer of two ricin-like proteins (RCA_60_) [11,12]. Since the agglutinin itself is much less toxic than ricin, RCA_120_ is used in diverse studies as a ricin simulant or a plant lectin. Otherwise, laboratory works dealing with deadly toxins need high-level attention for safety management.

On the other hand, in vivo assays are still widely used in Asian developing countries including Japan though an alternative in vitro approach is craved. Extensive efforts are underway on this issue using, for instance, fluorescence labeled peptide fragments [13], enzyme-linked immunosorbent assay (ELISA) [14], fluorescence resonance energy transfer (FRET) adopted-endopeptidase [15,16], and mass spectrometry [4]. Lateral flow immunoassays [17,18] which use anti-BoNT antibodies bound on nanobeads seem to provide rapid sensing approaches. Cell-based assays [19], immuno-sorbent surface area assays (ALISSA) [20], and binding and cleavage (BINACLE) assays [21,22] are proposed as highly sensitive sensing approaches.

**Figure 2:**
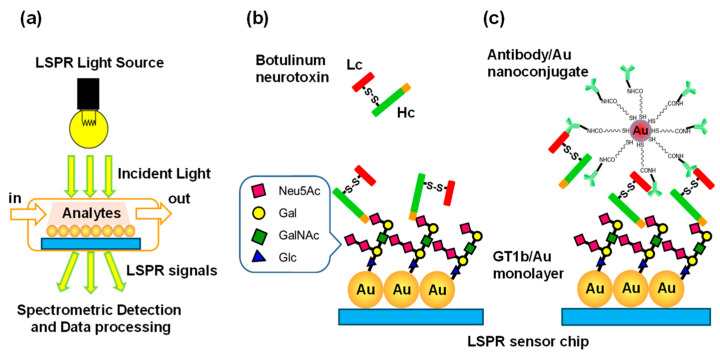
LSPR sensing approaches to AB toxins using a sensor chip coated with human glycolipids. (**a**) The sensor chip is incorporated in a flow LSPR sensing system, which has a UV/Vis spectrometer and a cuvette holder [21,22]. (**b**) A biosensor chip for BoNTs coated with GT1b-Cer glycolipids. The human glycolipid is immobilized on a tiny glass substrate and used to capture the target toxins on the glass surface discriminating their heavy chains (Hc) (B-subunit of AB toxins). (**c**) An advanced sensing approach to AB toxins, which utilizes a multivalent IgG antibody/Au nanoconjugate together with the biosensor chip. Those test samples which may contain BoNTs or other AB toxins are treated with this multivalent IgG/Au conjugate in advance to block their Lc domains acting as a main body of toxins. The target toxins are trapped on the sensor chip in a sandwiched manner and analyzed in the flow LSPR sensing system. A part of the illustration was adapted with permission from reference [23]. Copyright 2021 American Chemical Society.

In our research project, we try to develop such a portable biosensor that enables us to perform ‘on-site’ detection for biological toxins. Assuming a scene polluted by biological toxins, we applied an LSPR sensor system [23,24,25,26]. Our LSPR approach is characterized by the use of glycolipid sensor chips (Figure 2a,b) that carry a certain class of human cell-membrane glycolipids on the surface. There, human glycolipids act as artificial ligands to capture a targeted AB toxin on the sensor chip. We assembled different kinds of glycolipid chips starting from scratching the surface of a tiny glass plate literally in the manner of ‘from scratch’ [23,24,25,26]. The Au nanoparticles being spread on the glass plate also play an essential role in the LSPR sensing system [27]. We have already observed that the LSPR sensing system enables us a rapid detection for certain kinds of biological toxins including BoNT/A in their discriminative manners [23,24,25,26]. On the other hand, detection sensitivity in a range of 50~100 ng/mL is insufficient when the high toxicity of BoNTs and other AB toxins are considered. Another concern can come up in those laboratory works which need to handle or target neurotoxins in their intact, very toxic forms. From the viewpoints of sustainability and safety management, an aspect of a simple and safe approach should be introduced there. In the present study, we expect that these concerns may be solved in a combinatorial use of immunological tools which have shown enormous developments and applications in recent decades [28]. In this paper, we demonstrate an advanced sensing approach to AB toxins, which is based on their unique quaternary protein structures. In practice, multivalent antibody/Au nanoconjugates, which are prepared from IgG antibodies and Au nanoparticles, are applied in a pretreatment process for the test samples which may contain a target toxin. After the A-domain of the target toxins is blocked, the test sample is analyzed in the flow LSPR sensing system equipped with the glycolipid biosensor (Figure 2c).

## 2. Results and Discussion

### 2.1. Design of Multivalent IgG Antibody/Au Nanoconjugates

**Figure 3:**
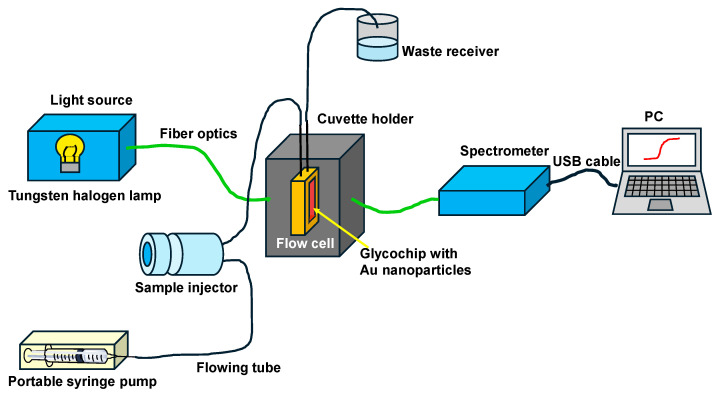
A flow LSPR sensing system used in the present study. The flow LSPR sensing system is assembled of two major parts. One is for flow, sample injection, and waste receiver. The second is the optical LSPR sensing system composed of UV-VIS light source (tungsten-halogen lamp), cuvette holder, photodetector, and PC processor. The cuvette holder has a room for setting a glycolipid/Au glass plate for use as biosensor chip.

**Scheme 1:**
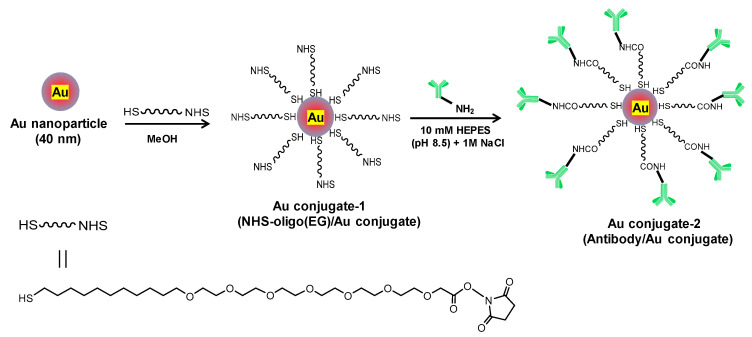
Preparation of multivalent antibody/Au conjugates using an oligo-ethylene glycol (EG) alkanethiol linker (HS-(CH_2_)_11_-EG_6_-OCH_2_-COONHS).

**Scheme 2:**
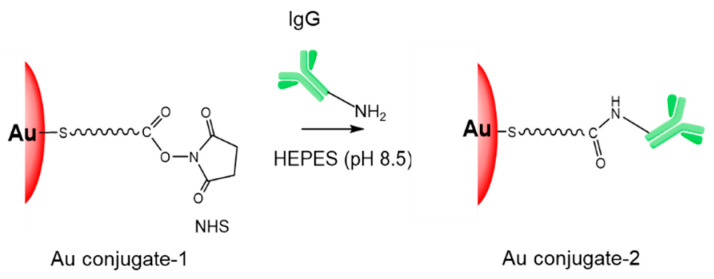
Conjugation reaction between IgG Lys-amino groups and NHS-activated carboxylic acids on Au nanoparticles to prepare multivalent IgG/Au nanoconjugate (Au conjugate-2).

As shown in Figure 2, the proposed LSPR detection system makes use of monoclonal IgG antibodies against the A-domain of AB toxins acting as a main body of toxins possessing enzymatic activity. The IgG antibody is installed on Au nanoparticles and used together with a human cell-membrane glycolipid. We have already established a main frame for the flow LSPR sensing approach to a biological toxin system using human cell-membrane glycolipids as biosensor chips in preceding studies [23,24]. The flow LSPR sensing system in Figure 3 needs no change in the machinery hardware from those we previously reported. An appropriate choice for the anti A-domain antibody followed by its installation on the surface of Au nanoparticles becomes a key point of the present approach. Thanks to vast progress in immunological studies and the associated protein engineering [29], various sorts of monoclonal IgG antibodies are commercially available. We selected monoclonal IgG antibodies with specificity to the A-subunit of RCA_60_ and RCA_120_ as well as those to the Lc domain of BoNTs from commercially available sources. Each of these IgG antibodies was immobilized on the surface of Au nanoparticle (40 nm ϕ) with a chemical method (Scheme 1). The Au surface was conjugated in advance with an NHS-activated oligo-(EG) linker in methanol suspension. This linker carries a thiol (SH) group at the exo-terminus of alkyl chain (C_11_), which covalently bonds to Au in methanol to afford the Au nanoparticle (Au conjugate-1)-carrying NHS groups. The NHS group on the other side reacts with Lys ε-amino groups in the IgG protein to afford the antibody/Au conjugate (Au conjugate-2). This reaction is conducted in HEPES buffer (pH 8.5) with conditions analogous to Schotten–Bauman reactions (Scheme 2).

**Figure 4:**
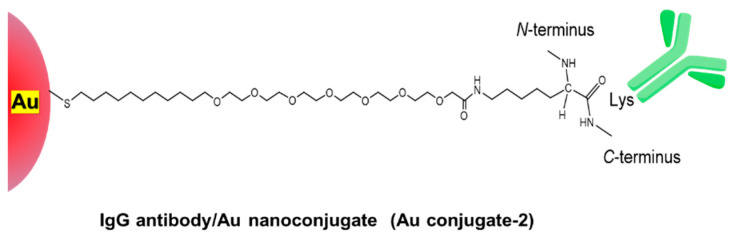
A local structure of antibody/Au nanoconjugate (Au conjugate-2) around an amide linkage between the oligo-EG linker and Lys in the antibody Fc domain.

Various types of NHS-activated biochemicals are also available in commercial sources and have been applied in marking reactions of IgG antibodies with molecular tags [30]. According to technical notes opened by biochemical industries, one IgG antibody accepts 3 or 4~7 fluorescent probes in the reaction with an NHS-activated FITC reagent without losing its inherent antigen-binding activity [31]. IgG antibodies have a conserved region rich in lysine (l-Lys) residues near the *C*-terminus of the antibody fragment crystallizable region (Fc) [32]. The NHS-activated Au surface is likely to react with one of these Lys residues in the Fc domain to afford the antibody/Au nanoconjugate possessing a covalent amide linkage as illustrated in Figure 4. Lys rarely appears around the antigen binding fragment (Fab) idiotype region of IgG proteins [33], and this fact helps chemical approaches for marking target IgG antibodies with fluorescence, enzyme, or other molecular tags. In the present study, the NHS oligo-(EG) linker was applied in excessive amount in the initial conjugating reaction with Au nanoparticles (Section 3). Provided that the surface of Au nanoparticle (40 nm ϕ) is fully coated with the NHS EG linker, one Au nanoparticle can receive approximately 20 molecules of IgG antibody on the surface. In this calculation, the molecular size of an IgG antibody (150 kDa) is assumed to have a diameter size at 15 nm ϕ and an occupied area at 180 nm^2^. Though ambiguity remains in the position of the Lys amide linkage and the resulting molecular dimension, the Au surface carries multiple IgG antibodies to constitute the multivalent IgG/Au nanoconjugate with a macromolecular structure as illustrated in Scheme 1.

Au nanoparticles with different diameter sizes and shapes are also available in commercial sources. The present study used the Au nanoparticle in 40 nm ϕ size in conjugation reactions. Ag-Cu particles might be in another choice considering their high LSPR potentials [34]. Au nanoparticles are physicochemically more stable, less toxic, and thus seemed easier to handle than the Ag-Cu ones. Au nanoparticles modified with biomolecules in this size can make a stable colloidal suspension in buffers, which is of high significance in our intended LSPR sensing approach to AB toxins. We have noticed in the present study that all the antibody/Au conjugates can be easily prepared by chemical processes like those by solid-phase synthesis. In addition, all the products can maintain the deep and vivid red color characteristic of Au colloidal solutions for a couple of days when stored in a refrigerator (4 °C).

**Scheme 3:**
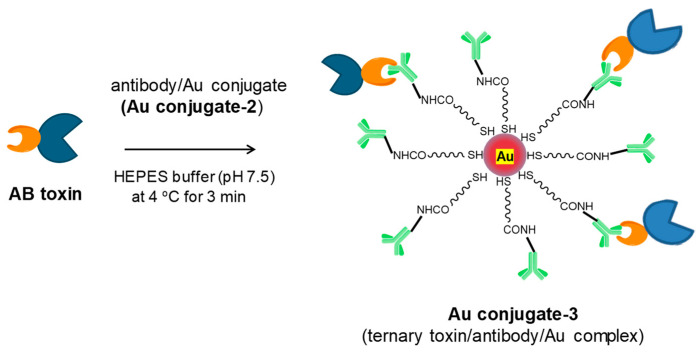
A pretreatment process for each test sample with the multivalent IgG antibody/Au nanoconjugate prior to flow LSPR sensing analysis. Before being applied in the flow LSPR sensing system, a test sample containing a target AB toxin is mixed with a multivalent antibody/Au conjugate (Au conjugate-2) in HEPES buffer to block the A-subunit of the target toxin in the form of a ternary toxin/antibody/Au complex (Au conjugate-3).

There are two different methods of using the multivalent antibody/Au nanoconjugate. One is to use it after a test sample is applied in LSPR sensor. In this case, the target toxin is already captured on the glycolipid chip as illustrated in Figure 2b and then reacts with this conjugate in a flow reaction to trap the toxin in a sandwiched form (Figure 2c). The other method is to use it before injection into the flow LSPR system as shown in Scheme 3. A test sample is mixed with the multivalent IgG antibody/Au conjugate (Au conjugate-2) in advance, and the pretreated sample is analyzed. In the second method, the target toxin is captured by the IgG antibody/Au conjugate in the sandwiched form making a ternary toxin/antibody/Au complex (Au conjugate-3). The second method is expected to better align with our aiming, rapid, and safe sensing approach than the first one. That is because the treated test samples can be detoxified by the multivalent antibody/Au nanoconjugate prior to analyses, though the degree of detoxification needs certification. This approach is, however, more challenging than the first one. The large ternary complex needs to land on the glycolipid chip in a short time and maintain a tight adhesion with the human glycolipid for tens of minutes (10~20 min) to acquire LSPR signals for sensing. Moreover, their interactions should take place in a species-specific fashion without being affected physically by the complexation with the multivalent IgG/Au conjugate.

### 2.2. LSPR Detection of RCA_120_ Using the Multivalent Antibody/Au Nanoconjugate

First, we evaluated the proposed LSPR approach using RCA_120_ as a homologue of ricin (Figure 1). The flow LSPR sensing system (Figure 2 and Figure 3) was set up for the detection of RCA_120_ as summarized in Table 1, in which the kind of IgG antibody, the diameter size of the Au nanoparticle, and the kind of human glycolipid installed on the sensor chip are shown. In addition to the target AB toxins (RCA_120_ and ricin), negative controls are also listed together with their concentrations.

RCA_120_ was dissolved in HEPES buffer at three different concentrations (10, 5, and 1.0 ng/mL) and used as a positive control in place of ricin. BSA and influenza virus hemagglutinin were tested as negative controls at higher concentrations (100 ng/mL) than those of the target toxin. Each of these test samples was pretreated with the multivalent IgG/Au nanoconjugates (Scheme 3) and injected into the flow LSPR sensing system as described in Section 3.

**Figure 5:**
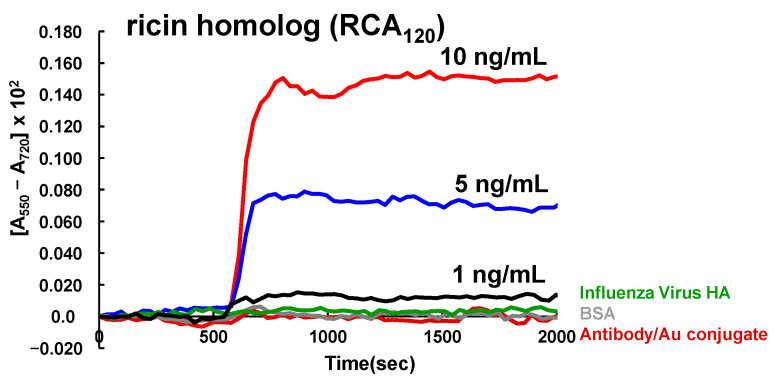
LSPR sensorgrams of RCA_120_ measured at three different concentrations after the pretreatment process of mixing with the multivalent IgG/Au nanoconjugate to block the A-subunit acting as a main body of biological toxin. RCA_120_ is used as a less toxic substitute of ricin (RCA_60_). Sensorgrams of negative controls (influenza hemagglutinin, BSA, and antibody/Au conjugate) measured at ca. 100 ng/mL are also shown. After the pretreatment process, each test sample (250 μL) was injected at 500 s (waiting time) and then delivered with running HEPES buffer at 4 mL/h and processed on a PC software (Section 3).

The results are shown with LSPR sensorgrams in Figure 5, which indicate that the proposed approach enables us to perform a quick and species-selective detection for RCA_120_ with sensitivity in a range of 1.0~5.0 ng/mL and sensing time within 25 min. The sensitivity is enhanced approximately by 20 times compared to our former approach without using the pretreatment process [25]. The sensorgrams also indicate that the multivalent antibody/Au nanoconjugate-2 makes no LSPR response by itself. BSA and influenza virus hemagglutinin (A/H1N1/New Caledonia) also show negligible response even if they are applied in larger amounts (100 ng/mL) than RCA_120_ (1~10 ng/mL). These data support our expectation that the pretreatment process of the RCA_120_ with the multivalent antibody/Au nanoconjugate exerts no damage in the flow LSPR sensing approach based on the use of the Lac-Cer glycolipid sensor chip. Instead, sensitivity is greatly increased by the IgG/Au conjugate used in the pretreatment process. The amplification of the LSPR signal is thought to result from an interphase Au/Au interaction [26] which is caused by tight adhesion of the toxin/IgG/Au ternary complex on the glycolipid sensor chip (Figure 2).

### 2.3. LSPR Detection of BoNTs in Different Serotypes (A, B, and E)

Encouraged by the preceding result, the proposed sensing approach was further evaluated with *C. botulinum* neurotoxins in three different serotypes, which are denoted as BoNTs/A, B, and E (Table 2). These three BoNT serotypes are reported to cause serious disorders in neuron-transmitting systems while showing some differences in antigenicity and pathogenicity to each other [4]. Throughout experiments, they were handled carefully following the safety management described in Section 3. The amounts of BoNT/A and B were set at 1.0 ng/mL and less than that as shown in Table 2 (entries 1 to 8). For BoNT/E, the amount was increased up to 5 ng/mL. Different from the preceding LSPR sensing system for RCA_120_ and ricin, the human glycolipid was replaced with GT1b-Cer. The multivalent antibody/Au conjugate was changed to those carrying IgG antibodies against their Lc chains corresponding to the A-subunit of ricin. The size of Au nanoparticles on the sensor chip was also changed from 20 nm ϕ to 40 nm ϕ according to our preceding optimization study [23,24].

**Figure 6:**
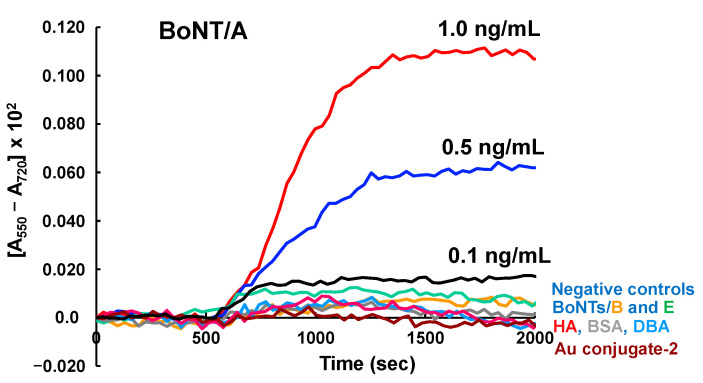
LSPR sensorgram of BoNT/A after pretreatment with the multivalent IgG antibody/Au nanoconjugate. The other serotypes (B and E) of BoNTs are also tested as one of the negative controls as listed in Table 2 and show together with the target BoNT/A. In this experiment, each test sample was treated with the multivalent conjugate in advance and injected into the flow LSPR system with a waiting time of 500 s and flow rate of 4 mL/h (Section 3). Typical LSPR data are shown when measurements were repeatedly conducted (Table S1, Supplemental Materials).

The proposed LSPR sensing system was tested twice or three times for BoNT/A. A typical LSPR sensorgram is shown in Figure 6, and other experimental results are compiled in Table S1 in Supplementary Materials. These data indicate that the LSPR responses against BoNT/A are almost linearly changed by concentrations in the range between 0.1 ng/mL and 1.0 ng/mL. The sensitivity is amplified by 500~1000 times compared to our previous approach (50 ng/mL for BoNT/A/Hc [25] and 100 ng/mL for BoNT/C [23]). Excluding the time required for the pretreatment process and the waiting time before sample injection (Section 3 and Scheme 3), the flow LSPR detection for one sample is completed within 25 min. LSPR responses from the other serotypes of BoNTs (BoNT/B at 1.0 ng/mL and BoNT/E at 10 ng/mL) are almost undetectable. These results show the utility of the present flow LSPR system with respect to conducting a serotype-selective detection for BoNT/A out of A, B, and E serotypes. The sensorgrams in Figure 6 also indicate that the other proteins used as negative controls make no detectable LSPR response even when applied in large amounts (ca. 100-fold in excess of BoNT/A). We checked this result by conducting experiments three times for BSA as the negative control (Table S1, Supplementary Materials). Concerning other proteins, a spike protein (HA) of influenza virus (A/H1N1, New Caledonia) makes no detectable response though this protein and has a receptor binding domain for sialo (Neu5Ac) bioconjugates expressed in mammalian cells. This is because of the high selectivity of both IgG and GT1b-Cer to the target toxin. In addition, a hydrophobic Au surface is covered with polar glycosyl chains of GT1b-Cer. All these results support and justify the concept of using the multivalent antibody/Au nanoconjugate in our LSPR sensing system (Figure 2c).

**Figure 7:**
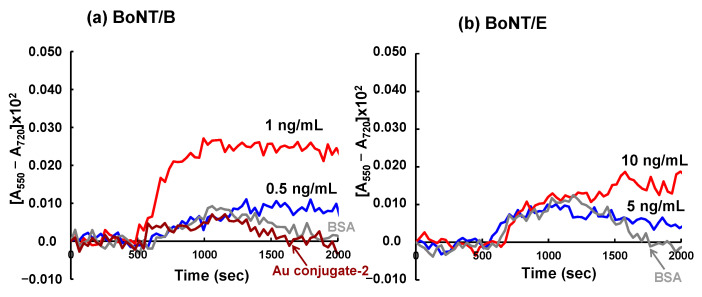
LSPR sensorgram of (**a**) BoNT/B and (**b**) BoNT/E after treatment with the multivalent IgG/Au nanoconjugate of the corresponding anti (BoNT/Lc chain) IgG antibody (entries 7~10, Table 2). The two serotypes were equally analyzed with injection time of 500 s and flow rate of 4 mL/h (Section 3). Typical LSPR data are shown when measurements were repeatedly conducted (Table S1, Supplemental Materials).

Figure 7a shows the LSPR sensorgrams of BoNT/B measured using anti (BoNT/B/Lc chain) antibody and GT1b-Cer sensor chip (entries 7 and 8, Table 2). This result shows BoNT/B is detectable in the rage of 0.5~1.0 ng/mL concentration. Sensitivity is, however, apparently decreased from the previous case of BoNT/A detection (Figure 6). A more serious decrease is observable in the LSPR analysis of BoNT/E (Figure 7b). These results mean that the LSPR responses from BoNTs are remarkably changed by serotypes. When the minimal concentration required for LSPR sensing is compared, detection sensitivity changes in the following order: BoNT/A (0.1~0.5 ng/mL) > BoNT/B (0.5~1.0 ng/mL) > BoNT/E (>10 ng/mL). From a practical viewpoint, the present LSPR approach needs optimization for each of IgG antibody and glycolipid chip to gain a clear response from the E serotype of BoNT. Otherwise, the present LSPR sensing condition may mislead a false-negative test result in the diagnosis.

The enhanced LSPR responses from RCA_120_ and BoNT/A are of those successful cases when both the IgG antibody and the human glycolipid are correctly applied in the sensing system, whereas the low response from the BoNT/E serotype represents the case when one of them is incorrectly selected. Provided that monoclonal IgG antibodies are correctly installed, the result of BoNT/E means that this serotype has already lost a binding affinity to GT1b. This assumption is partially applicable in the LSPR response of BoNT/B which was poorer than that of BoNT/A (Figure 6 and Figure 7). Conversely, the proposed LSPR sensing system depends on the specificity of two kinds of interactions: one is between the antibody and its antigen, and the other between the receptor protein and its ligand. We assume that the combinatorial use of these two different interactions will provide us with a novel LSPR sensing approach which can discriminate the quaternary protein structure of a target AB toxin.

### 2.4. Probable Reasons Why LSPR Responses Are So Different Among the Three Serotypes of BoNTs

BoNTs secreted by *C. botulinum* in soils or foods are known to form complexes with non-toxic hemagglutinins (HA) and non-HA proteins [4]. Though the complexation occurs in the light chain (Lc) of BoNTs away from receptor binding sites in the heavy chain (Hc), it may trigger an allosteric effect, affecting the inherent activity of the Hc. In our experiments, we used such native BoNTs that were purified from the culture of *C. botulinum* and supplied by a Japanese chemical industry (Section 3). Judging from their molecular weights, i.e., BoNT/A (500 KDa), B (500 KDa), and E (300 KDa), the A and B serotypes undergo complexation with both HA and non-HA proteins, whereas the E serotype makes a smaller complex with either HA or non-HA only. Figure 6 and Figure 7 seem to reflect the difference in complexation among the three serotypes.

The Hc chains of BoNTs have a pair of binding sites for establishing the tight adhesion on host neurocytes, leading to internalization through the membrane [4,6,7]. One is for poly(sialo) gangliosides like GT1b-Cer enriched in mammalian neurocyte membranes, and the other for the membrane-bound glycoprotein called synaptic vesicle protein 2 (SV2) [4,6,7]. The cooperativity of the dual binding sites is not made clear as far as we know. There is a similar case in many carbohydrate-binding proteins including lectins like RCA_120_ and selectins [35,36], which usually possess dual or an extended binding site to establish multivalent and cooperative interactions with glycolipids and glycoproteins. The existence of such dual binding sites is thought to endow BoNTs and other AB toxins with a flexibility in host recognition and an enhanced binding affinity to target cells as well. The complexation with non-toxic HA and/or non-HA proteins is thought to cause a shift in the power balance of the dual binding sites in different ways among the three types of BoNTs to give the LSPR sensorgrams as shown in Figure 6 and Figure 7. Provided the power balance of BoNT/E is shifted to the glycoprotein (SV2), we can rationalize the poor LSPR response of this serotype.

There is another probability. The complexation with non-toxic HA proteins can totally change the inherent activity of BoNTs into those for recognizing the other classes of gangliosides such as GD1a, GD1b, and GQ1b in place of GT1b-Cer or even asialoglycoproteins without Neu5Ac. The HA proteins also have dual carbohydrate-binding domains for accepting various kinds of carbohydrate motifs including Neu5NAc and GalNAc [37]. This fact means that complexation with HA proteins can change a mode of host cell recognition in a competitive manner to the dual binding sites in Hc domains.

Another factor may occur in the pretreatment process. The process of blocking the Lc chain of BoNT with the multivalent IgG/Au conjugate (Scheme 3) may affect the inherent activity of the Hc chain of the target BoNT. This assumption is closely related to the principle of the advanced LSPR sensing approach using the multivalent conjugate to reduce the toxicity of AB toxins in advance. In the present study, we used those monoclonal IgG antibodies that are specific to each of the BoNTs Lc chains. They are modified with the NHS oligo-EG alkanethiol linker, which is conformationally flexible and chemically reactive with Lys ε-amino groups in IgG Fc chains, and installed on the surface of Au nanoparticles (Scheme 2 and Figure 4). The potential utility of the IgG antibody/Au nanoconjugate is shown in the analyses of RCA_120_ and BoNT/A (Figure 5 and Figure 6). In this context, the unexpected result does not seem to arise from the pretreatment process with the multivalent antibody/Au nanoconjugate.

In the present LSPR sensing system, we assumed that GT1b-Cer could serve as a common receptor ligand of BoNTs and work out almost equally to the three serotypes of toxins (A, B, and E). As discussed above, this assumption was invalid due to several reasons. We may have overlooked an important cause other than the presence of the dual receptor binding sites in Hc domains and the complexation with the two non-toxic proteins in Lc domains. This working hypothesis, however, has provided us with many important insights into our LSPR sensing approach.

### 2.5. Features of the Proposed LSPR Sensing Approach in Comparison with Other Reported Approaches

**Figure 8:**
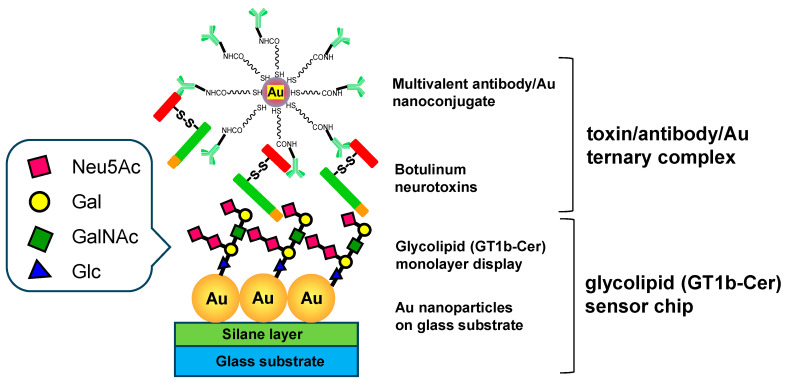
An LSPR sensor chip carrying human cell membrane glycolipid (GT1b-Cer) and its notable function to capture and determine a target botulinum neurotoxin in combination with a multivalent IgG antibody/Au nanoconjugate. A part of the illustration was adapted with permission from ref. [26]. Copyright 2023 American Chemical Society.

Our LSPR sensing approaches are based on the use of human cell-membrane glycolipids for detection of biological toxins. In the present study, we tested the advanced approach in which the multivalent IgG antibody/Au nanoconjugate is used in advance to downregulate the toxicity of AB toxins. In general, multivalent ligand molecules like IgM antibodies are thought to possess an enhanced binding affinity to target antigens compared to monoclonal IgG antibodies. An analogous enhancement is known to occur in carbohydrate/protein interactions [35,36] and is rationalized in terms of ‘multivalent binding effect’ [38] and ‘cluster effect’ [39]. These effects are incorporated in the molecular design of artificial glycoconjugates with different structural forms like glycopolymers and glycolipids [35,36,38,39,40,41]. In the present study, the human glycolipid and the IgG antibody are installed separately on the surface of Au nanoparticles expecting that they can exert multivalent effects in the flow LSPR sensing system (Figure 8). Except for the case of BoNT/E, the proposed LSPR system worked out as expected. The use of these two multivalent Au conjugates can enhance detection sensitivity and selectivity if an appropriate selection is made for the glycolipid sensor chip and the IgG antibody. The sensing system is based on a molecular-based mechanism, in which each of the A and B subunits is recognized selectively. The target toxin is trapped and detoxified eventually on the glass chip in a sandwiched manner (Figure 8). Though extensive verification and validation studies are still needed, the results of RCA_120_ and BoNT/A and B (Figure 5, Figure 6 and Figure 7) accord with our aiming sensing method.

The use of the flow sensing system is also characteristic of our approach (Figure 3). The present LSPR system is assembled within portable size and weight [24]. The time required for the analysis of one sample is shorter than 30 min when the sample is pretreated with the multivalent IgG/Au conjugate in advance. The rapid detection is one of the advantageous points over the others reported in the literature as listed in Table 3. Generally, protein/glycan interactions take place in a rapid and species-specific manner at the expense of binding affinity compared to protein/protein and antibody/antigen interactions. The LSPR sensorgrams in Figure 5 and Figure 6 indicate that the binding event on the glycolipid chip quickly establishes an equilibrium state and saturates in a few minutes. The flow LSPR sensing system is based on such quick and selective toxin/glycolipid interactions. In addition, the multivalent ligand expression on both the IgG/Au and the glycolipid/Au conjugates are advantageous for shifting the equilibrium toward association to accelerate analyses. Au nanoparticles used in these conjugates are defined in size in our previously reported manner [24,26] as shown in Table 1 and Table 2. These Au nanoconjugates themselves play key roles in the present approach not only for preparing the multivalent ligands in a similar way to solid-phase syntheses but also for the phenomenon of localized surface plasmon resonance. We expect that the multivalent antibody/Au nanoparticles are also useful with respect to capturing a target toxin and increasing its apparent concentration. In addition, the trapping process should work for the decontamination of AB toxins in test samples and match the safety management for those laboratory works dealing with neurotoxins as well.

There are different detection or identification methods for biological toxins, in which strong efforts have been devoted in the research works dealing with neurotoxins produced by *C. botulinum*, *C*. *tetanus* toxins, and other environmental bacteria in *Clostridium* family [4]. Thanks to these precedent efforts, different BoNT detection tools are available as listed in Table 3. Including our LSPR approaches (entries 10~12), each tool seems to have both advantages and disadvantages over the others with respect to sensitivity and required time. Safety, cost-effectiveness, and simplicity are also important items. In addition, sustainable approaches on the side of kindness to animals and the environment are becoming more important than those on the side of efficiency. Every living thing favors sugar and utilizes glycoconjugates in forms of glycolipids and glycoproteins. Pathogenic bacteria and viruses take proactive approaches to human glycoconjugates by expressing adhesive proteins, spike proteins, and sometimes expressing proteinous toxins. Our LSPR sensing approach is based on these sugar/protein interactions occurring in nature on the human cell-membrane surface.

## 3. Materials and Methods

### 3.1. Reagents and Instruments

All chemicals and biomolecules were purchased from biochemical companies and used without further purification. The oligo-ethylene glycol (EG) alkanethiol linker (HS-(CH_2_)_11_-EG_6_-OCH_2_-COONHS) [42] was from ProChimia Surfaces (Sopot, Poland). Monoclonal antibodies for the three BoNTs/Lc (mouse IgG2a for type A, mouse IgG1 for type B, and mouse IgG for type E, each of which is specific to the corresponding serotype) were from R & D Systems (Minneapolis, MN, USA). Anti-RCA_60_ monoclonal antibody (mouse IgG1), which recognizes the A-chain of RCA_60_ and RCA_120_ was from GeneTex (Irvine, CA, USA). Lectin from horse gram (*Dolichos biflorus*) was purchased from EY Laboratories (San Mateo, CA, USA). Ganglioside GT1b was from IsoSep AB (Tullinge, Sweden). Influenza virus hemagglutinins (HA, H1N1/New Caledonia) were purchased from ITSI-Biosciences (Johnstown, PA, USA). *R. communis* agglutinin (RCA_120_) was purchased from Vector Laboratories Inc. (Burlingame, CA, USA). Recombinant-free, native botulinum toxins in serotypes of A, B, and E (BoNT/A, B, and E) with an origin of bacterial cultivation were purchased from Wako Pure Chemical Industry (Osaka, Japan) before unification in FUJIFILM Wako Pure Chemical Corporation (Osaka, Japan). A biosafety cabinet (LAD-1000XAS, Class II) was purchased from Labconco Corporation (Kansas, MO, USA). Lac-Cer and GT1b-Cer derivatives possessing a lipoic amide group were prepared in our previously reported way [23,24]. Au nanoparticles (20 and 40 nm ϕ) were supplied from Tanaka Precious Metals (Tokyo, Japan).

Microwave irradiation was conducted with a Wave Magic MWO-1000S (2.45 GHz, EYELA Co., Ltd., Tokyo, Japan). A flow LSPR sensing system was assembled in a prototype in collaboration works [23,24]. Its main machinery units like a tungsten halogen lamp (LS-1), 400 μm core diameter optical fibers (P400-1-UV/VIS), and a spectrometer (QE65000 or QE65 Pro, bandwidth: 475−851 nm) with a long-path filter (OF1-GG475) were purchased from Ocean Optics (Dunedin, FL, USA). The other assemblies like flow pump (TE-361N, Terumo, Tokyo, Japan), Peltier-controlled cuvette holder (qpod) with Z-height of 15 mm (Quantum Northwest, Liberty Lake, WA, USA), sample injector (Rheodyne 9725 or 7725, IDEX Health & Science LLC, Rohnert Park, WA, USA), and flow cell (FLAB50-UV-02, GL Science, Tokyo, Japan) were also from commercial sources and incorporated in the flow LSPR sensor system in the prototype.

### 3.2. Preparation of Lac-Cer and GT1b-Cer Glycolipid Chips

The glass chip was ornamented with Au nanoparticles in a defined size (Figure 2) in the manner of those previously reported [23,24]. Briefly, a tiny glass plate (W 26 mm × D 12 mm × H 2.0 mm) was purchased from GL Sciences (Tokyo, Japan), cleaned with Piranha solution (H_2_O_2_/H_2_SO_4_ = 1/3, *v*/*v*) at 80 °C for 40 min, and treated with 10% aminopropyltrimethoxysilane (APTMS) in ethanol for 15 min. Caution: A piranha solution reacts violently with organic materials and should be handled with extreme care. The glass plate was washed gently with ethanol and dried at 50 °C for 3 h under vacuum. After cooling, the plate was treated with 8.3 mM *N*-hydroxysuccinimide (NHS)-activated lipoic acid in DMF (2 mL) under microwave irradiation (100 W, 70 °C, 600 s) and N_2_ atmosphere. The glass substrate was activated with lipoic acid, fixed with a handmade silicon mold, and coated with Au nanoparticles with a defined size on one side of surfaces.

Each Lac-Cer and GT1b-Cer was installed on the Au-activated glass substrate under microwave irradiation in our previously reported manner [23]. Briefly, the Au-activated glass plate was immersed in a methanol solution of either Lac-Cer or GT1b-Cer (49 μM, 2 mL), and the installation was carried out with a microwave reactor. The microwave irradiation (250 W) was conducted under N_2_ atmosphere on a PC program to run an on/interval/off sequence for controlling an adequate temperature inside using a cooling device (experimental condition C3 in ref. [23] for Lac installation) or without using a cooling device (experimental condition B2 in ref. [23] for GT1b installation). The obtained GT1b-Cer or Lac-Cer glycochip was thoroughly washed with methanol and water. If they still show an unspecific response to BSA or other negative proteins, they are prewashed with a 1 mM methanol solution of lipoic acid.

### 3.3. Preparations of Multivalent Antibody/Au Nanoconjugates (Scheme 1)

To a methanol suspension of Au nanoparticles (40 nm ϕ, 1 mL, 1.3 × 10^11^ particles), 100 μL of an NHS activated oligo-EG linker (HS-(CH_2_)_11_-EG_6_-OCH_2_-COONHS, 1.5 mg/mL in methanol) was added, and the reaction mixture was shaken gently at 37 °C overnight (8~10 h). After centrifugation (15,500× *g*, 8~15 min) at 4 °C and removal of supernatant solutions, the residual Au nanoparticles were dispersed in 10 mM HEPES buffer (pH 8.5) containing 1 M NaCl, sonicated for a few minutes, and again centrifugated. After repeating the washing process further twice, Au conjugate-1 bearing NHS-oligo-EG linker on the surface was dispersed in 10 mM HEPES buffer (pH 8.5) containing 1 M NaCl and immediately used in the next step to prepare the multivalent antibody/Au nanoconjugate (Au conjugate-2, Scheme 1).

Each of the monoclonal IgG antibodies against the A-subunit of RCA_60_ (0.5 mg/mL, 2 μL) or the Lc chain of BoNTs/A, B, and E (0.5 mg/mL, 5 μL) was added to the colloidal suspension of the tagged Au nanoparticles (Au conjugate-1) in HEPES buffer (pH 8.5) (20 or 30 μL). The reaction mixture was shaken at 550 rpm and 4 °C with Thermo-shaker overnight. The colloidal suspension of the derived antibody/Au nanoconjugate (Au conjugate-2) was centrifuged (15,500× *g*, 4 °C) for 8 min (RCA_120_ antibody/Au conjugate) or 15 min (BoNTs antibody/Au conjugate). After removal of the upper supernatant, Au nanoparticles deposited at the bottom phase were again suspended in a 10 mM HEPES buffer (pH 7.5) containing 150 mM NaCl. The washing process was repeated at least twice. The deposited multivalent IgG antibody/Au nanoconjugate (Au conjugate-2) was suspended with a 10 mM HEPES buffer (1.0 mL) and kept in a fridge at 4 °C until use. The red color characteristic of Au colloidal solutions lasted for at least a couple of days throughout the present research work.

### 3.4. Preparations of Test Samples for LSPR Sensing Analysis

RCA_120_ was dissolved in HEPES buffer (pH 7.5) at four different concentrations (10, 5.0, 1.0, and 0.0 ng/mL) to prepare the test samples of LSPR sensing. Similarly, test samples containing three serotypes of botulinum neurotoxins, i.e., BoNT/A (1.0, 0.5, 0.1, and 0.0 ng/mL), BoNT/B (1.0, 0.5, and 0.0 ng/mL), and BoNT/E (10, 5.0 and 0.0 ng/mL), were prepared. The concentration of 0.0 ng/mL means that test samples without toxins (0.0 ng/mL) were prepared for checking possible LSPR responses from multivalent IgG/Au nanoconjugates in the absence of target toxins. For the E-serotype of BoNT, test samples with increased concentrations (5.0 and 10.0 ng/mL) were prepared. This was due to its poor LSPR response, which will be discussed in the Section 2. To examine unselective binding interactions with other adhesive proteins, samples containing either BSA, DBA lectin, or influenza virus (A/H1N1) hemagglutinin (HA) were prepared. Their concentrations were fixed at 100 ng/mL, which was 10~1000 times higher than those of target toxins.

Prior to LSPR analysis, every test solution was treated with the multivalent IgG antibody/Au nanoconjugate (Au conjugate-2, Scheme 1) bearing a monoclonal IgG antibody against an A-subunit domain. For instance, the sample solution of BoNT/serotype-A (0.5 ng/mL, 1 mL) was mixed with Au conjugate-2 (15 μL) in 10 mM HEPES buffer (pH 7.5) at 4 °C for 3 min and applied in the following LSPR analysis as a pretreated test sample.

### 3.5. A Flow LSPR Sensing System

The flow LSPR sensing system has a mechanism of detecting the wavelength shift of incident UV/vis light at 550 and 720 nm. The shift is caused by interactions with analytes on the biosensor chip. In the present system, Au nanoparticles spread on the glass chip are responsible for causing the optical phenomenon of localized surface plasmon resonance [27] and making the wavelength shift by a close interaction with target toxins and other macromolecules on their surface. A flow LSPR sensing system was assembled in collaboration works [23,24] and operated on a PC software item (OOIBase32 software (Ocean Optics, version 2.0)). An optical system is composed of conventional UV/vis spectrometer and cuvette holder, and a flow delivery system is made up of a syringe pump, a sample injector, and a waste receiver as illustrated in Figure 3.

The running buffer (10 mM HEPES buffer (pH 7.5) plus 150 mM NaCl) was filtered with a 0.22 μm PTFE membrane filter (Merck Millipore, Tullagreen, Ireland) and degassed before use. For RCA_120_ detection, 250 μL of the pretreated sample solution (Au conjugate-3, ternary toxin/antibody/Au complex, Scheme 3) was injected through an injector and delivered with the running buffer for 3.75 min at a flow rate of 66.6 μL/min. In cases of BoNTs (A, B and E) detection, each was injected with 1 mL of their pretreated sample solutions and delivered for 15 min at 66.6 μL/min. In every case, the sample injection was initiated after the baseline of the LSPR sensorgram became stable. Usually, LSPR signals appeared soon after the sample injection and reached saturation in several minutes to give LSPR sensorgrams for 25 min. The LSPR sensing was conducted setting an integration period at 8 msec and the number of data acquisitions or accumulation times at 2000 times. The obtained data were processed on OOIBase32 software (Ocean Optics, version 2.0).

### 3.6. Safety Management

BoNTs/A, B, and E are toxic if inhaled or injected. Though RCA_120_ is much less toxic, every biological toxin must be handled very carefully conforming to rules and regulations that are announced publicly in individual institutes. In the present study, all the experiments handling biological items were conducted with the approval of the Ministry of Health, Labour and Welfare of Japan. Every test sample, glass syringe, glycolipid chip, and plastic tube was thoroughly washed with aqueous sodium hypochlorite or processed thermally with autoclave. These works were performed in designated rooms in our institutes conforming to all the measures and regulations for the physical containment of biohazards [43].

## 4. Conclusions

Our goal is to establish a rapid and safe detection method for those biological toxins that may be abused in crimes and other illegal actions. As far as we have tested with RCA_120_, the botanic toxin, ricin is detectable at 1.0 ng/mL concentration within 30 min with the proposed LSPR sensing system. BoNT/A and BoNT/B are detectable in the rage of 0.1~0.5 and 0.5~1.0 ng/mL, respectively. Compared to our preceding LSPR approach, sensitivity is increased more than 10 times owing to the combinatorial use of the multivalent IgG/Au nanoconjugate. This achievement may be still insufficient when compared to the mouse in vivo assays which are based on the potent neurotoxicity of BoNTs. There is, however, a greater advantage in our approach from the perspectives of simplicity and safety management for laboratory works. We expect that the multivalent IgG/Au nanoconjugates not only detoxify AB toxins in advance but also enhance apparent concentrations on the Au surface to increase detection sensitivity and selectivity.

During this study, we encountered unexpected results; LSPR responses largely changed among BoNT A, B, and E serotypes in the order A > B > E when GT1b-Cer was installed on the sensor chip. The E serotype of BoNT made no clear response below 5 ng/mL as far as examined in the present study and seemed to require more than 10 ng/mL for clear detection in the present system. We discussed possible reasons and causative factors for these results and have suggested that their affinity to GT1b-Cer may be modulated by a power balance or cooperativity for the dual acceptor binding sites, one of which recognizes the human synaptic vesicle glycoprotein (SV2). In addition, natural BoNT proteins from *C. botulinum* permit complexation with non-toxic HA and non-HA proteins. The power balance may be shifted by these non-toxic proteins.

The proposed LSPR sensing approach, which started from scratching the surface of a tiny glass plate, is showing slow but steady progress. There are still many things to do in the direction of practical applications such as a validation study using, for example, real food samples and a verification study from the perspective of safety management. We will continue this approach with a robust vision to establish such a definitive sensing method that can discriminate the unique quaternary protein structure of biological toxins in a rapid, safe, and selective manner.

## Figures and Tables

**Table 1:** An LSPR sensing system for *R. communis* RCA_120_ as a homologue of ricin (RCA_60_).

Entry	LSRP Sensing System			Test Samples	
	Target Toxins	Antibody on Au Nanoparticle (Size) ^a^	Glycolipid on Au Nanoparticle (Size) ^a^	Positive Control (ng/mL)	Negative Control (ng/mL)
1	RCA_120_ or ricin (RCA_60_)	RCA_60_/A-chain on Au (40 nm ϕ)	Lac-Cer/Au (20 nm ϕ)	RCA_120_ (10 ng/mL)	BSA ^b^ (100 ng/mL)
2				RCA_120_ (5.0 ng/mL)	Influenza HA ^c^ (100 ng/mL)
3				RCA_120_ (1.0 ng/mL)	Antibody/Au complex ^d^

^a^ Given in diameter (ϕ nm). ^b^ Bovine serum albumin. ^c^ Hemagglutinin of influenza virus (A/H1N1/New Caledonia). ^d^ Anti (RCA_60_ A-subunit) IgG antibody/Au conjugate free of toxin complex (Au conjugate-2 in Scheme 1).

**Table 2:** LSPR sensing system for *C. botulinum* neurotoxins (BoNTs).

Entry	LSRP Sensing System			Test Samples	
	Target Toxins	Antibody on Au Nanoparticle (Size) ^a^	Glycolipid on Au Nanoparticle (Size) ^a^	Positive Control (ng/mL)	Negative Control (ng/mL)
1	BoNT/A	BoNT/A/Lc ^b^ on Au (40 nm ϕ)	GT1b-Cer/Au (40 nm ϕ)	BoNT/A (1.0 ng/mL)	BoNT/B (1.0 ng/mL)
2				BoNT/A (0.5 ng/mL)	BoNT/E (10 ng/mL)
3				BoNT/A (0.1 ng/mL)	BSA ^c^ (100 ng/mL)
4					DBA ^d^ (100 ng/mL)
5					Influenza HA ^e^ (100 ng/mL)
6					Antibody/Au complex ^f^
7	BoNT/B	BoNT/B/Lc on Au (40 nm ϕ)	GT1b-Cer/Au (40 nm ϕ)	BoNT/B (1.0 ng/mL)	BSA ^c^ (100 ng/mL)
8				BoNT/B (0.5 ng/mL)	Antibody/Au complex ^f^
9	BoNT/E	BoNT/E/Lc on Au (40 nm ϕ)	GT1b-Cer/Au (40 nm ϕ)	BoNT/E (10 ng/mL)	BSA ^c^ (100 ng/mL)
10				BoNT/E (5.0 ng/mL)	

^a^ Size is given with diameter (nm ϕ). ^b^ An antibody against a light chain (Lc) of BoNT. ^c^ Bovine serum albumin. ^d^ Herbal lectin in horse gram (*Dolichos biflorus*) with affinity to α-d-GalNAc. ^e^ Hemagglutinin of influenza A virus (H1N1, New Caledonia). ^f^ Anti (BoNT/A or B serotype/Lc) IgG antibody/Au conjugates free of toxin complex (Au conjugate-2 in Scheme 1).

**Table 3:** A brief comparison between mouse in vivo bioassays and our LSPR methods for detection of BoNTs.

Entry	Methods	Sensitivity (ng/mL)	Duration	Ref.
1	Mouse in vivo assay	0.01~0.1	1~4 days	[4]
2	LSPR (monolayer style) ^a^	50	<30 min ^b^	[23,25]
3	LSPR (double layer style) ^c^	5.0	<30 min ^b^	[26]
4	LSPR (present study in sandwich style)	0.1~1.0 ^d^	<30 min ^b^	this study

^a^ Au nanoparticles (40 nm ϕ) are arrayed on a sensor chip in a homogeneous monolayer form. ^b^ LSPR detection time required for one test sample. ^c^ Au nanoparticles (60 nm + 40 nm ϕ) in two different sizes are applied in a heterogeneous double-layered form on the sensor chip. ^d^ Sensitivity for BoNT/A (0.1~0.5 ng/mL) and BoNT/B (0.5~1.0 ng/mL).

## Data Availability

Some data presented in this study are available upon request from the corresponding author.

## Supplementary Materials

The following supporting information can be downloaded at: https://www.mdpi.com/article/10.3390/ijms252413352/s1.
