# Supplementary material for: An Advanced Sensing Approach to Biological Toxins with Localized Surface Plasmon Resonance Spectroscopy Based on Their Unique Protein Quaternary Structures
Source: Int J Mol Sci. 2024 Dec 12;25(24):13352. doi: 10.3390/ijms252413352 (PMC11677682; doi:10.3390/ijms252413352)
Supplement: Supplementary file 1 [file ijms-25-13352-s001.zip › ijms-3224310-supplementary.pdf]

Supplementary Materials

An Advanced Sensing Approach to Biological Toxins with Localized Surface Plasmon Resonance Spectroscopy Based on their Unique Protein Quaternary Structures

Hirotaka Uzawa [1*], Satoshi Kondo [1], Takehiro Nagatsuka [1], Yasuo Seto [2] and Yoshihiro Nishida [3]

[1] Nanomaterials Research Institute, National Institute of Advanced Industrial Science and Technology (AIST), 1-1-1 Higashi, Tsukuba, 305-8565, Japan

[2] Forensic Science Group, RIKEN SPring-8 Center, Sayo-gun, Hyogo 679-5148, Japan

[3] Division of Applied Biological Chemistry, Graduate School of Environmental Horticulture, Chiba University, Matsudo, Chiba 271-8510, Japan

Table S1. Summary of experiments and results of LSPR response of BoNT serotypes as target
toxins and BSA as the negative control examined at different concentrations.

BoNT/A/Lc/antibody-Au nanoconjugates

Test samples	Concentration ng/mL	LSPR response x 10^2	Number of experiments	Mean response x 10^2	Standard deviation x 10^2
BoNT/A	1.0	0.105<br>0.0775	2	0.09125	0.0194
	0.5	0.061<br>0.050<br>0.055	3	0.055	0.0055
	0.1	0.0169	1	(0.0169)	
BSA	100	0.00187<br>0.00625<br>-0.00125	3	0.00344	0.0038

BoNT/B/Lc/antibody-Au nanoconjugates

Test samples	Concentration ng/mL	LSPR response x 10^2	Number of experiments	Mean response x 10^2	Standard deviation x 10^2
BoNT/B	1.0	0.0246	1	(0.0246)	
	0.5	0.0087	1	(0.0087)	
BSA	100	0.003<br>0.005	2	0.004	0.0014

BoNT/E/Lc/antibody-Au nanoconjugates

Test Samples	Concentration ng/mL	LSPR response x 10^2	Number of experiments	Mean response x 10^2	Standard deviation x 10^2
BoNT/E	10.0	0.0143<br>0.00625	2	0.010	0.0057
	5.0	0.004	1	(0.004)	
BSA	100	0.00375<br>0.00063	2	0.002	0.0022

LSPR response: A mean value of the intensity of LSPR sensorgram in range of 1750 ~ 2000 sec.
